# Human Leukocyte Antigen C*12:02:02 and Killer Immunoglobulin-Like Receptor 2DL5 are Distinctly Associated with Ankylosing Spondylitis in the Taiwanese

**DOI:** 10.3390/ijms18081775

**Published:** 2017-08-16

**Authors:** Chin-Man Wang, Sheng-Hung Wang, Yeong-Jian Jan Wu, Jing-Chi Lin, Jianming Wu, Ji-Yih Chen

**Affiliations:** 1 Department of Rehabilitation, Chang Gung Memorial Hospital, Chang Gung University College of Medicine, Taoyuan 33375, Taiwan; cmw1314@cgmh.org.tw; 2 Institute of Stem Cell and Translational Cancer Research, Chang Gung Memorial Hospital at Linkou, Taoyuan 33375, Taiwan; shawnwang@cgmh.org.tw; 3 Department of Medicine, Division of Allergy, Immunology and Rheumatology, Chang Gung Memorial Hospital, Chang Gung University College of Medicine, No. 5, Fu-Shin St. Kuei-Shan, Taoyuan 33375, Taiwan; yjwu1962@adm.cgmh.org.tw (Y.-J.J.W.); jingchilin@gmail.com (J.-C.L.); 4 Department of Veterinary and Biomedical Sciences, Department of Medicine, University of Minnesota, St. Paul, MN 55108, USA; jmwu@umn.edu

**Keywords:** ankylosing spondylitis, natural killer cell, human leukocyte antigen C (HLA-C), killer immunoglobulin-like receptor (KIR)

## Abstract

Human leukocyte antigen (HLA) class I ligands and Killer immunoglobulin-like receptors (KIRs) regulate the cytolytic activity of natural killer (NK) cells and certain T cells. We examined their genetic predisposition to disease susceptibility and clinical phenotypes in Taiwanese ankylosing spondylitis (AS) patients. KIR genotyping and Human Leucocyte Antigen C (HLA-C) sequencing were performed in 653 Taiwanese AS patients and 952 healthy controls. KIR genotype distributions and HLA-C allele frequencies were compared in patients and controls and among patients with and without HLA-B27 positivity, early age onset and spinal syndesmophytes. HLA-C alleles were functionally characterized using 3D structural modelling with peptide simulation. This study discovered that the HLA-C*12:02:02 allele (43.42% vs. 3.31%; *p* < 0.00001 odds ratio (OR), 16.88; 95% confidence intervals (CI): 11.27–25.28) confers a strong risk for Taiwanese AS development. The 3D modelling results identified four unique amino acid polymorphisms, Ala73, Trp156, Arg219 and Met304, that may affect the function of the HLA-C*12:02:02 allele. KIR2DL5 (*p* = 0.0047; *p_FDR_* = 0.0423) and the KIR Bx haplotype (*p* = 0.0000275) were protective against Taiwanese AS, while KIR 2DS4/1D (22 base pair truncated deletion; *p* = 0.0044; *p_FDR_* = 0.1998) appeared to be a risk factor for it. KIR2DL5 combined with the HLA-C1/C2 heterozygous genotype showed a protective effect (AS 5.97% vs. normal 11.66%; *p* = 0.002; *p_FDR_* = 0.0127, OR, 0.48 95% CI: 0.33–0.70); in contrast, KIR 2DS4/1D combined with the HLA-C1C1 homozygous genotype (AS 45.33% vs. normal 35.92%; *p* = 0.002; *p_FDR_* = 0.0127, OR, 1.48 95% CI: 1.21–1.81) represented a risk factor for AS development. Our data suggested that interactions between KIRs and their cognate HLA-C ligands may contribute to the pathogenesis of AS.

## 1. Introduction

Ankylosing spondylitis (AS), a prototypical chronic immune-mediated inflammatory arthritis disorder of spondyloarthritides (SpA), is a highly familial, heritable disease as shown by strong evidence from genetic association twin and family studies [1,2,3]. Multiple genetic interactions have been implicated in the aetiology of AS disease and present with heterogeneous manifestations [4,5,6]. The genetic transmission and patterns of inheritance in AS are highly complex. Therefore, there is immense interest for identifying the genetic factors in the AS complex disease entity.

Natural killer (NK) cells dynamically interact via inhibitory surface receptors with human leukocyte antigen (HLA) ligands to acquire a “licensing” process, and express functional competence during development [7]. Licensed NK cells with self-MHC (Major Histocompatibility Complex) specific receptors are more readily activated and more responsive than unlicensed NK cells without self-MHC-specific receptors [8]. NK cell activity reflects the balance between inhibitory receptors specific for MHC class I molecules and activating receptors with diverse specificities. The elevated frequency of killer immunoglobulin-like receptor (KIR) 3DL1 (Three Ig Domains and Long cytoplasmic tail 1) expressing NK cells in SpA patients may contribute to a reduction in IFN-γ production [9]. NK cells interact with fibroblast-like synoviocytes (FLS), leading to proinflammatory responses with increased IL-15 expression by FLS followed by the production of proinflammatory chemokines, cytokines, and matrix metalloproteinases (MMPs) in SpA and rheumatoid arthritis (RA) patients [10]. However, the precise role of NK cells in the pathogenesis of SpA remains unclear, as conflicting data require further clarification.

KIRs (killer immunoglobulin-like receptors) regulate the cytolytic activity of NK cells and certain T cells through binding to HLA class I ligands [11]. KIRs comprise critical inhibitory receptors to ensure self-recognition, which dampens NK cell activation upon interaction with cognate MHC class I ligands [11]. Human NK cells subjected to NK cell licensing highlight the potential functional influence of KIR and HLA genes in disease as well as inter-individual differences in NK cell potency [12,13]. Specific HLA Class I molecules may bind and trigger cell surface receptors specified by KIR genes that regulate the physiological functions of NK cells [13]. Thus, it is important to identify relevant associations between the KIR functional genotype that contributes to AS susceptibility and the development of clinical characteristics.

The *KIR* gene clusters show extensive genetic diversity, as do the HLA Class I loci, which encode ligands for KIR molecules. The activating *KIR* genes and their corresponding HLA ligand groups show strong negative correlations across populations with distinct evidence for coevolution [8,14]. The inhibitory receptor and autologous HLA interactions impact cell function and demonstrate that the resting human NK repertoire is highly attuned but variegated in the immune response [14]. Increasing evidence from epidemiological studies reveals that particular *KIR* and HLA genotypes are associated with certain human disease outcomes, although the functional explanation for these associations is poorly understood. The interaction of HLA-C with KIR plays the dominant role to control human NK cell response [15]. In addition, HLA-C alleles appear to associate with AS susceptibility [16,17,18]. Herein, we aimed to investigate the association of HLA-C alleles, KIR functional genes and their interaction in the genetic predisposition to disease susceptibility and clinical phenotypes in Taiwanese patients with AS.

## 2. Results

### 2.1. Clinical Characteristics in the Taiwanese Ankylosing Spondylitis (AS) Cohort

This study recruited 653 Taiwanese AS patients (535 males and 118 females) with a mean age at disease onset of 25.18 ± 9.27 years old, as shown in Table 1. Of 653 AS patients, 94 patients showed disease onset at less than or equal to 16 years (early onset AS), while 559 did not. A total of 601 (92.04%) patients were positive for HLA-B27, and 308 patients demonstrated syndesmophyte formation on spinal X-ray examination, including 137 patients with modified Stoke’s Ankylosing Spondylitis Spinal Score (mSASSS) < 24 and 303 patients with mSASSS ≥ 24.

### 2.2. Association of Human Leukocyte Antigen (HLA) Class I Ligand (HLA-C*12:02:02) with Taiwanese AS

The current study successfully performed HLA-C allele discrimination in 653 AS patients and 952 normal controls using sequencing-based genotyping. We identified 39 HLA-C alleles in the Taiwanese population. The HLA-C*12:02:02 allele frequency (AS 43.42% vs. normal 3.31%) was significantly higher in AS patients than in normal controls (*p* < 0.00001 Odds Ratio (OR), 16.88; 95% Confidence Interval (CI): 11.27–25.28). In contrast, other alleles, including HLA-C 01:02:01 (*p* = 9.01 × 10^−6^, OR, 0.45 95% CI: 0.31–0.64), HLA-C 03:02:01 (*p* = 9.01 × 10^−6^, OR, 0.45 95% CI: 0.31–0.64) and HLA-C 03:04:01 (*p* = 9.01 × 10^−6^, OR, 0.45 95% CI: 0.31–0.64), revealed protective effects in Taiwanese AS patients (Table 2). Notably, 542 of 546 HLA-C*12:02:02 allele-positive (22 homozygous and 524 heterozygous) individuals also carried HLA B27, indicating an interactive association. NK cell education largely depends on KIRs that recognize HLA-C epitopes distinguished by a dimorphism designated as a C1 (asparagine) and C2 (lysine) group based on the amino acid difference at position 80. When stratified by C1 and C2 epitopes, a higher frequency of HLA C1 epitope carriers was observed in AS patients (91.3%) than in normal controls (85.82%) (*p* = 1.844 × 10^−6^ OR, 1.761; 95% CI: 1.396–2.222), as shown in Table 3. Next, we compared allele frequencies between the AS patients with each characteristic and the AS patients without these characteristics (gender, syndesmophyte formation, HLA-B27 positivity and age at disease onset ≤16 years). No additional distribution differences were observed with regard to AS clinical phenotypes.

### 2.3. Association of Killer Immunoglobulin-Like Receptor (KIR) Genes with Taiwanese AS Susceptibility and Clinical Characteristics

KIRs and their ligand pathways play an important immune regulatory role during antigen presentation. We applied an allele-specific genotyping method for nine *KIR* genes with different variation ratios to characterize the genotypes. We first performed univariate analyses and multivariate logistic regressions on each of the *KIR* genetic variables for AS susceptibility. Interestingly, our study used a large cohort of samples and paradoxically found that *KIR*2DL5 was significantly less common in the Taiwanese AS patient group than in the control group (*p* = 0.0047; *p_FDR_* = 0.0423 OR, 0.75 95% CI: 0.61–0.91). In contrast, the *KIR* 2DS4/1D genotype was identified as a risk factor for AS, although this association was insignificant after multivariate analysis (*p* = 0.0044; *p_FDR_* = 0.1998 OR, 1.23 95% CI: 1.01–1.5). We next investigated whether *KIR* gene variations had different genetic background effects in AS phenotypes. As shown in Table 4, in the case-only analysis, no statistically significant distribution differences were observed between individual *KIR* genes and AS clinical characteristics. Thus, our data indicate that ethnic differences in *KIR* distribution have no significant associations with Taiwanese AS clinical phenotypes.

### 2.4. Haplotype Construction of KIR Genes in Taiwanese AS Patients and Healthy Controls

Haplotype construction and the analysis of *KIR* genes in Taiwanese AS patients and healthy controls were performed, with two broadly classified *KIR* groups (AA and Bx). We then investigated whether the *KIR* haplotypes affected AS. The *KIR* AA haplotype showed a significant risk association with Taiwanese AS development (AS 50.15% vs. normal 40.44%; *p* = 0.0000275). In contrast, the *KIR* Bx haplotype showed a protective role in Taiwanese AS subjects (AS 48.85% vs. normal 59.56%; *p* = 0.0000275). These findings suggest that *KIR* haplotypes with predominant inhibitory functional receptors play regulatory roles in AS pathogenesis that contributes to disease development. Again, we did not observe any significant associations between *KIR* haplotypes (AA and Bx) and AS clinical characteristics. Thus, the inhibitory dominant KIR haplotypes do not contribute to the development of other clinical phenotypes and complex disease characteristics of AS.

### 2.5. Association of KIRs and HLA-C1/C2 Epitope Combinations with Taiwanese AS Susceptibility

Genetic variation in KIRs is responsible for the complex gene effects impacting NK cell function, while HLA class I molecules tightly regulate cytotoxic T lymphocyte responses. We speculated that the specific combinatorial effect of *KIR* genes and their HLA-C ligands may be implicated in the outcome of AS. As shown in Table 5, we found that the frequency of AS in the *KIR*2DL5 combined HLA-C1/C2 heterozygous genotype was significantly lower than that in normal controls (AS 5.97% vs. normal 11.66%; *p* = 0.002; *p_FDR_* = 0.0127, OR, 0.48 95% CI: 0.33–0.70). In contrast, the frequency of AS in the *KIR*2DS4 combined HLA-C1C1 homozygous genotype was significantly higher than that in normal controls (AS 45.33% vs. normal 35.92%; *p* = 0.002; *p_FDR_* = 0.0127, OR, 1.48 95% CI: 1.21–1.81). These results suggest that interactions between KIRs and their cognate HLA class I ligands play a crucial role in the pathogenesis of AS.

### 2.6. Structural Features of Specific Major Histocompatibility Complex (MHC) Class I HLA-C*12:02:02 Alleles Might Be Associated with AS in Taiwanese Patients

**Figure 1:**
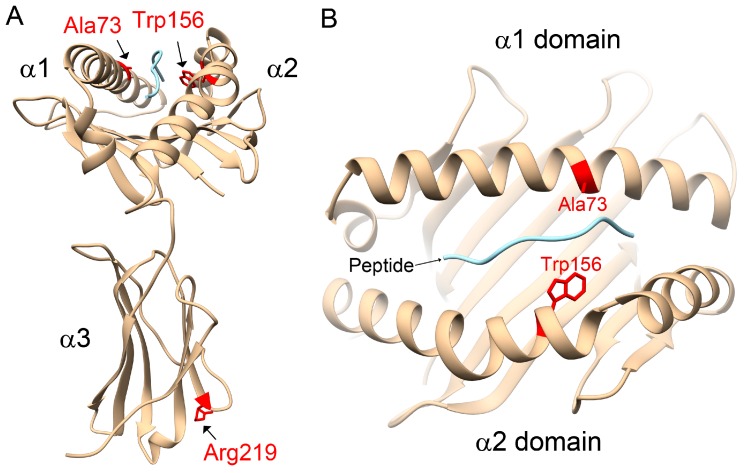
3D Modeling of the α1, α2 and α3 domains of human HLA-C*12:02:02 in complex with peptide. (**A**) The residues colored in red represent the unique amino acid polymorphisms Ala73, Trp156 and Arg219 of HLA-C*12:02:02 compared to HLA-C*01:02:01 and HLA-C*03:02:01. (**B**) View from the top of (**A**). The Ala73 and Trp156 polymorphisms are located in the peptide-binding groove between the α1 and α2 domains and interact with the peptide presented by HLA-C*12:02:02.

Similar to the HLA-A and HLA-B groups, the heterodimer proteins of HLA-C alleles are composed of an α chain (heavy chain) and a β chain (β-2 microglobulin). The α chain consists of α1 and α2 domains, forming a peptide-binding groove between the two domains, followed by the α3 domain and a transmembrane helix in the carboxyl end of the HLA protein (Figure S1). We compared the amino acid sequences of HLA-C*12:02:02 with two other common alleles in the HLA-C family, HLA-C*01:02:01 and HLA-C*03:02:01 (Table 2). As shown in Figure S1, there were four amino acid polymorphisms, Ala73, Trp156, Arg219, and Met304, found to be unique in HLA-C*12:02:02 compared to HLA-C*01:02:01 and HLA-C*03:02:01. Among them, Ala73 and Trp156 are located within the α1–α2 domains, whereas Arg219 is located within the α3 domain, and Met304 is located near the end of the C-terminal transmembrane helix (Figure S1). Furthermore, the 3D structure of human HLA-C*12:02:02 containing the α1, α2 and α3 domains was modeled (Figure 1), as described in the Methods section. The locations of the three unique amino acid polymorphisms, Ala73, Trp156 and Arg219, within the α1–α3 domains of HLA-C*12:02:02 are colored red in Figure 1. Importantly, the Ala73 and Trp156 polymorphisms are located in the peptide-binding groove between the α1 and α2 domains and interact with the peptide presented by HLA-C*12:02:02 (Figure 1B), suggesting that Ala73 and Trp156 in HLA-C*12:02:02 might affect the binding affinities, orientations, or conformations of the peptides presented in the groove.

## 3. Discussion

Genetic differences in MHC alleles, especially HLA-B27, confer the greatest risk to AS but are not the only significant genetic factor driving the disease development process. The highly polymorphic KIRs modulate NK and T cell actions against target cells through their interactions with HLA-C ligands. The current study shows that specific *KIRs*, HLA Class I genes and their combinations under natural selection result in the diverse characteristics of individuals and populations, and contribute to AS pathogenesis in the Taiwanese population [19,20]. As a whole, we observed that in the Taiwanese population, the *KIR* AA haplotype, HLA-C1202 allele and HLA-C1 epitope confer risk for AS development, while *KIR*2DL5, *KIR* Bx and HLA-C2 have a protective effect.

KIRs are involved in the activation/inhibition of NK cells through their interaction with MHC class I, particularly on target cells. KIRs and MHC class I ligands, the HLA-B group (particularly HLA-B27) and possibly other HLA molecules have been associated with differential NK cell activity and function, adding to the growing evidence for the involvement of KIRs in AS disease development [21]. Previous studies have demonstrated that *KIR*3DL1/*KIR*3DS1, 2DL5 and *KIR*2DS1 are risk factors in the pathogenesis of AS [21,22]. A positive association of KIR3DS1 (activating receptor) and a negative association of KIR3DL1 (inhibitory receptor) with AS development have been suggested. Nevertheless, neither the *KIR* gene content of particular *KIR* haplotypes nor *KIR*3DL2 polymorphisms contribute to AS in Caucasian (UK) patients [23,24]. *KIR*3DS1 segregates as an allele with a short cytoplasmic tail and characterizes activation receptors that are expressed by a higher percentage of NK cells in *KIR*3DS1 homozygous donors than in heterozygous donors [25]. *KIR*3DS1 shows an increased frequency association in combination with HLA-B alleles carrying Bw4-I80 in the trans position in Spanish and Azoreans populations with AS, compared with B27 controls, whereas KIR3DL1 was decreased in AS patients [26,27]. KIR3DS1 or KIR3DL1 in combination with the HLA-B27s/HLA-B Bw4-I80 genotypes may modulate the disease development of AS [28]. The activation of either NK or T cells via the KIR3DS1 receptor may represent a critical event in AS development, while the presence of the functional KIR3DL1 receptor confers a protective effect. In Asians, studies with small sample sizes have demonstrated that the frequency of 3DL1/3DL1 is decreased, while that of 3DL1/3DS1 is increased in Chinese and Thai AS populations [21,29]. As a consequence, a general imbalance mediated by protective/inhibitory and risk/activating allotypes from specific *KIR* genotypes is responsible for AS susceptibility. With the genetic background of HLA-B27, variation at the KIRs and their corresponding specific HLA-C ligands may contribute to the pathogenesis of AS. Both HLA-C and HLA-B are located at the MHC cluster on Chromosome 6. We found HLA-C1202 allele had strong linkage with HLA-B27 positivity. However, their influence on the ability of NK cells and T cells to recognize and lyse targets in the immune response and the functional effects of KIR polymorphisms remain largely unknown [26,27,28,29,30].

*KIR* gene variations with activating effects might associate with susceptibility to AS by influencing NK cell activity once HLA-C2 epitope ligands are present [16,17,18,21]. It has been proposed that the orientations or conformations of peptides, even the conformational flexibility of HLA proteins, might affect the association of HLA subtypes with AS [31]. However, in opposition to the literature, our study observed that the HLA-C1 epitope was a risk factor for AS, especially the HLA-C*12:02:02 allele containing the HLA-C1 epitope. Compared with other HLA-C alleles, 3D structural modelling of the highest-frequency allele in AS patients, HLA-C*12:02:02, showed different protein-peptide interactions resulting from the binding affinities, orientations, or conformations during peptide presentation on Ala73 and Trp156. Although the precise underlying mechanisms remain to be determined, we speculate that it may be related to differential NK cell activity.

KIR2DL5 exhibits distinct inhibitory capacities through recruiting both SHP-1 and SHP-2, and its inhibitory capacity is more similar to that of the cytoplasmic domain of KIR2DL4 than that of KIR3DL1 [32]. Notably, our study comprised a large cohort of samples and paradoxically identified that KIR2DL5 was significantly less prevalent in the Taiwanese AS patient group than the control group (*p*_fishier_ = 0.004833). *KIR*2DL5 belongs to the *KIR* B haplotypes and shows associations with activating receptors related to high viral loads in primary human cytomegalovirus (HCMV) infection following HCMV-positive renal transplant, severe pandemic influenza A (H1N1) and dengue virus infections [32,33,34,35]. *KIR*2DL5 was shown to decrease the risk of systemic lupus erythaematosus (SLE), but increased the overall risk for viral infections in Japanese subjects [36]. However, our study had limitations, such as lacking genotyping for KIR2DL5A and KIR2DL5B (non-allelic expression form) at seven nucleotide positions that differentiate two functional genotypes characterizing KIR2DL5 protein expression [37,38]. *KIR*2DL5A is the predominant genotype in Asian populations, which is consistent with the results from our 282 commercially genotyped individuals [37]. *KIR* B haplotypes also showed a protective role for AS in our study, although the combinatory effects of activating KIRs are difficult to explain. Our study suggests that KIR2DL5 may serve as an independent KIR protective marker for Taiwanese AS, although the ligand is unknown [20].

KIR2DS4 was identified as a risk factor for AS based on a previous meta-analysis [22]. KIR*2DS4* presents as either a fully functional (KIR2DS4-*f*) or deleted non-functional (KIR2DS4/1D) variant with a 22-bp deletion in exon 5, due to a frame shift mutation and premature stop codon yielding a truncated KIR2DS4 protein with loss of the transmembrane and cytoplasmic domains [39]. Our data revealed that *KIR*2DS4 deletion carriers with low KIR2DS4 functional expression were at risk for AS. Additionally, our study showed that *KIR* A haplotypes with multiple *KIR* inhibitory genes represented risk factors, suggesting that this inhibitory regulatory receptor is critical for AS susceptibility.

These discrepant results indicate the complex diversification of *KIR* gene structure and human evolution by genetic selection over long-term environmental stimulation. The remarkable polymorphism of *KIR* and HLA genes warrants descriptive gene frequency studies in different populations. The diversity distribution of Asians, especially Taiwanese citizens, demonstrates a similar high frequency (>98.6%) in framework-specific *KIR* genes (2DL1, 2DL3, 2DL4, 3DL1, 3DL2) among normal subjects and AS patients, although we did not apply these results to functional analysis. Nevertheless, the present study extends the association and confirms the contribution of the *KIR* genes to AS susceptibility, with an imbalance between activating and inhibitory *KIR* genes seeming to influence susceptibility to AS [40].

Combinatorial analyses of *KIR* genes and their HLA-C ligands may reveal the interaction effects contributing to AS pathogenesis. We observed protective effects for *KIR*2DL5 with the heterozygous HLA-C1C2 ligand combination genotype in a multivariate analysis of Taiwanese AS development. Our results indicated that HLA-C was beneficial in *KIR*2DL5 AS carriers possessing at least one C2 allele, while the opposite was not true for homozygous C1 carriers (C1/C1), resulting in risk outcomes. In contrast, the *KIR*2DS4 deletion and HLA-C1C1 ligand combination was identified as a risk factor for AS pathogenesis. These results suggest that the KIR gene content in combination with the specific ligand may enhance the influence on AS development.

## 4. Materials and Methods

### 4.1. Characteristics of the Study Populations

The present study recruited patients who fulfilled the 1984 revised New York diagnostic criteria for AS. Radiographs of the cervical, thoracic and lumbar spine were examined by rheumatology specialists to evaluate syndesmophyte formations according to the modified Stoke’s Ankylosing Spondylitis Spinal Score (mSASSS). To ensure the accuracy of evaluation, two rheumatology specialists independently scored the syndensmophyte formations by blindly reading radiographs of AS patients to establish inter- and intra-reader reliability. The X-ray observations were further classified into three groups: Group 1 patients showed no spine erosion, sclerosis or syndesmophyte formation; Group 2 patients showed <4 fused syndesmophyte formations (mSASSS < 24); and Group 3 patients showed >4 syndesmophyte formations (mSASSS ≥ 24), as described previously [41]. For the comparisons in this study, 952 healthy blood donors (568 males and 384 females, mean age: 47.98 ± 9.97) were recruited. This study was approved by the ethics committees of Chang Gung Memorial Hospital (Institutional Review Board (IRB) approval number 104-7310B; 26 November 2015). All experiments were performed in accordance with relevant guidelines and regulations. All patients provided written informed consent according to the Declaration of Helsinki.

### 4.2. HLA-B27 Determination

HLA-B27 antigen positivity was determined by flow cytometry analysis and/or polymerase chain reaction (PCR) assays. Briefly, the whole blood samples were stained with BD^TM^ HLA-B27 kit with fluorescein-conjugated anti-HLA-B27 phycoerythrin-conjugated anti-CD3 antibodies that specifically bind to leucocyte antigen. Samples were then analyzed using a FACSCalibur flow cytometer and HLA-B27 software (Becton Dickinson, San Jose, CA, USA). In PCR assays, two sets of primers were used to amplify *HLA-B* gene with genomic DNA. The first set of primers detect human HLA-B27/B40/B55 while the second set of primer specifically target HLA-B27. Amplification of human β-globin gene in the same PCR reaction was included as an internal control.

### 4.3. Nucleic Acid Isolation

Genomic DNA was isolated from Ethylenediaminetetraacetic acid (EDTA) anti-coagulated peripheral blood using the Puregene DNA isolation kit (Gentra Systems, Minneapolis, MN, USA).

### 4.4. Genotyping of KIRs

The presence and absence of *KIR* genes (*KIR* gene profiles) were established using the PCR-SSP *KIR* genotyping kit (KIR Genotyping SSP Kit). *KIR*2DL1 (279), 2DL3 (281), 2DL4 (282), 3DL1 (278), 3DL2 (282), and 3DL3 (282) showed positive rates over 98.5%. Thereafter, nine KIR genes (KIR2DL2, 2DL3, 2DL5, 2DS1, 2DS2, 2DS3, 2DS4-full, 2DS4-deleted, 2DS5, and 3DS1) in the remainder of the samples were genotyped using an in-house PCR-SSP primer set, the results of which were consistent with commercial kits applied to the same 282 samples. PCR reactions were prepared at a volume of 10 µL containing 0.08 µL Taq DNA polymerase (5 U/L), 9 µL Ready PCR buffer and 1 µL DNA (40 ng/L). All of the KIR genes were amplified using an ABI 9700 PCR Cycler (Applied Biosystems, Forster City, CA, USA) under the following conditions: initial denaturation at 96 °C for 2 min, followed by 10 cycles of 15 s at 96 °C and 1 min at 65 °C, and 20 cycles of 15 s at 96 °C, 50 s at 61 °C, and 30 s at 72 °C, with a 4 °C hold. The PCR products were electrophoresed on a 2% agarose gel and then visualized under ultraviolet light.

### 4.5. HLA-C Genotyping and DNA Sequencing

Genotyping of the HLA-C allele was performed using a commercial sequencing-based typing (SBT) kit (Applied Biosystems, Forster City, CA, USA). HLA-C genotypes were assigned by the HLA SBT uTYPE 6.0 software (Applied Biosystems, Forster City, CA, USA).

### 4.6. Homology Modelling of Protein Structures for HLA-C Alleles 

The amino acid sequences, residue numbers and the sequence alignment of HLA-C alleles were searched for on the HLA nomenclature website (http://hla.alleles.org). The α chain of HLA-C*12:02:02 is a protein of 342 amino acids. Based on a sequence search using Basic Local Alignment Search Tool (BLAST) (https://blast.ncbi.nlm.nih.gov), the structural information for HLA-Cw3 (Protein Date Bank codes: 1EFX) [42] was selected as a template for homology modelling of HLA-C alleles, in which the identity between HLA-C*02:02:02 and HLA-Cw was 96% within 277 aligned amino acids (A.A. 2–278). A human self-peptide of sequence RRKWRRWHL derived from vasoactive intestinal peptide receptor type 1 (pVIPR) is a known peptide displayed by AS-associated HLA-B*27 [31]. This RRKWRRWHL peptide was also modelled in complex with the HLA-C alleles to indicate the peptide-binding site. Subsequently, the 3D structures of human HLA-C alleles with peptides were simulated by Modeller 9.12 [43], with the python scripts using the functions of the AUTOMODEL class. The Discrete Optimized Protein Energy (DOPE) method [43] was used to select the best model from the 10 initially generated models. The protein-peptide interactions were analyzed using HotLig [44]. The molecular models were rendered using Chimera [45].

### 4.7. Statistical Analysis

Frequency comparisons of HLA-C alleles, individual *KIR* genes, *KIR* haplotypes (group A and group B) and KIR/HLA-C pairs between patients and controls were made using logistic regression or the *χ*^2^-test, and Fisher’s exact test was applied when appropriate. Based on the risk allele identified, *p*-values, odds ratios (ORs), and 95% confidence intervals (CIs) were then calculated. To account for multiple testing corrections, false discovery rate (FDR)-corrected *p*-values were generated using an FDR correction method in the modified version of QVALUE software (http://genomics.princeton.edu/storeylab/qvalue/). To investigate the genetic association with clinical characteristics, we controlled for each of the clinical characteristics and performed stepwise logistic regression analyses.

## 5. Conclusions

Collectively, our data suggest that the HLA-C*12:02:02 allele and KIR2DL5 have distinctive functional roles in AS development. The present study provides new insight into the effects of KIR, HLA, and KIR-HLA combinations on the immune response of NK and specific T cells, and their contributions in Taiwanese patients with AS.

## Figures and Tables

**Table 1:** Clinical characteristics of Taiwanese ankylosing spondylitis (AS) and normal controls.

Characteristics	AS Case	Normal
	*N* = 653	*N* = 952
Sex, *N* (%)		
Male	535/653 (81.93%)	568/952 (59.66%)
Female	118/653 (18.07%)	384/952 (40.34%)
Age, Mean ± Std.	25.18 ± 9.27	47.98 ± 9.97
Syndesmophyte		
None	345/653 (52.83%)	
mSASSS * < 24	111/653 (17%)	
mSASSS ≥ 24	197/653 (30.17%)	
HLA-B27		
Negative	52/653 (7.96%)	
Positive	601/653 (92.04%)	
Age onset group		
≤16	94/653 (14.4%)	
>16	559/653 (85.6%)	

* mSASSS: modified Stoke’s Ankylosing Spondylitis Spinal Score.

**Table 2:** Association of the major histocompatibility complex class I molecules (MHC I) HLA-C alleles with Taiwanese AS.

Allele Type	AS	Normal	Logistic Regression (Unadjusted)		Logistic Regression (Adjusted for Age and Sex)	
			*p*-Value	OR * (95% CI **)	*p*-Value	OR (95% CI)
01:02:01	131 (10.03%)	359 (18.86%)	1.73 × 10^−11^	0.48 (0.39–0.59)	9.01 × 10^−6^	0.45 (0.31–0.64)
01:02:03	0 (0%)	1 (0.05%)	0.9688	0.00 (n/a ^1^)	0.9847	0.00 (n/a)
01:03	1 (0.08%)	8 (0.42%)	0.1081	0.18 (0.02–1.45)	0.1964	0.22 (0.02–2.21)
01:08	2 (0.15%)	1 (0.05%)	0.3820	2.92 (0.26–32.22)	0.4191	11.83 (0.03–4742.61)
02:02:02	14 (1.07%)	1 (0.05%)	0.0035	20.62 (2.71–156.96)	0.0357	15.92 (1.20–210.68)
03:02:01	79 (6.05%)	239 (12.55%)	0.96 × 10^−9^	0.45 (0.34–0.58)	5.84 × 10^−6^	0.37 (0.24–0.57)
03:03:01	34 (2.6%)	66 (3.47%)	0.1682	0.74 (0.49–1.13)	0.5977	1.19 (0.62–2.29)
03:03:04	1 (0.08%)	0 (0%)	0.9668	n/a	0.9853	n/a
03:04	0 (0%)	1 (0.05%)	0.9688	0.00 (n/a)	0.9811	0.00 (n/a)
03:04:01:01	69 (5.28%)	215 (11.29%)	8.59 × 10^−9^	0.44 (0.33–0.58)	0.0007	0.45 (0.28–0.72)
03:04:02	0 (0%)	1 (0.05%)	0.9688	0.00 (n/a)	0.9814	0.00 (0.00–I)
03:04:04	12 (0.92%)	16 (0.84%)	0.8129	1.10 (0.52–2.32)	0.7649	1.24 (0.30–5.13)
03:17	1 (0.08%)	2 (0.11%)	0.7962	0.73 (0.07–8.05)	0.0627	11.36 (0.88–146.66)
03:85	0 (0%)	1 (0.05%)	0.9688	0.00 (n/a)	0.9893	0.00 (n/a)
04:01:01:01	31 (2.37%)	88 (4.62%)	0.0012	0.50 (0.33–0.76)	0.5464	0.82 (0.43–1.57)
04:01:01:05	0 (0%)	1 (0.05%)	0.9688	0.00 (n/a)	0.9811	0.00 (n/a)
04:03	8 (0.61%)	36 (1.89%)	0.0037	0.32 (0.15–0.69)	0.1476	0.43 (0.14–1.35)
05:01:01:01	0 (0%)	2 (0.11%)	0.9707	0.00 (n/a)	0.9773	0.00 (n/a)
06:02:01:01	16 (1.23%)	55 (2.89%)	0.0023	0.42 (0.24–0.73)	0.1356	0.52 (0.22–1.23)
07:01:01:01	1 (0.08%)	6 (0.32%)	0.1898	0.24 (0.03–2.02)	0.3103	0.15 (0.00–5.86)
07:02	2 (0.15%)	1 (0.05%)	0.3820	2.92 (0.26–32.22)	0.7454	0.65 (0.05–8.85)
07:02:01:01	155 (11.87%)	355 (18.64%)	3.06 × 10^−7^	0.59 (0.48–0.72)	0.0321	0.70 (0.50–0.97)
07:02:01:02	10 (0.77%)	9 (0.47%)	0.2924	1.62 (0.66–4.01)	0.5698	1.59 (0.32–7.78)
07:04:01	3 (0.23%)	13 (0.68%)	0.0882	0.33 (0.10–1.18)	0.7573	0.78 (0.16–3.76)
07:66	1 (0.08%)	3 (0.16%)	0.5323	0.49 (0.05–4.68)	0.6070	0.51 (0.04–6.57)
08:01:01	82 (6.28%)	164 (8.61%)	0.0150	0.71 (0.54–0.94)	0.1434	0.70 (0.44–1.13)
08:01:02	0 (0%)	1 (0.05%)	0.9688	0.00 (n/a)	0.9786	0.00 (n/a)
08:03:01	1 (0.08%)	8 (0.42%)	0.1081	0.18 (0.02–1.45)	0.6890	0.56 (0.03–9.62)
12:02:01	5 (0.38%)	0 (0%)	0.9680	n/a	0.9738	n/a
12:02:02	564 (43.19%)	63 (3.31%)	0	22.21 (16.89–29.21)	0	16.88 (11.27–25.28)
12:03:01:01	7 (0.54%)	26 (1.37%)	0.0272	0.39 (0.17–0.90)	0.1145	0.35 (0.09–1.29)
12:10:01	1 (0.08%)	0 (0%)	0.9668	n/a	0.9849	n/a
14:02:01	29 (2.22%)	71 (3.73%)	0.0169	0.59 (0.38–0.91)	0.1330	0.57 (0.27–1.19)
14:03	2 (0.15%)	2 (0.11%)	0.7060	1.46 (0.21–10.37)	0.5144	0.50 (0.06–4.06)
15:02:01	42 (3.22%)	85 (4.46%)	0.0760	0.71 (0.49–1.04)	0.8108	0.93 (0.53–1.65)
15:05:01	1 (0.08%)	1 (0.05%)	0.7897	1.46 (0.09–23.33)	0.9029	0.63 (0.00–1086.11)
16:02:01	0 (0%)	1 (0.05%)	0.9688	0.00 (n/a)	0.9844	0.00 (n/a)
16:04:01	1 (0.08%)	1 (0.05%)	0.7897	1.46 (0.09–23.33)	0.7381	2.05 (0.03–137.91)
17:01:01:01	0 (0%)	1 (0.05%)	0.9688	0.00 (n/a)	0.9819	0.00 (n/a)

* OR: Odds Ratio, ** CI: Confidence Interval; ^1^ Not Available.

**Table 3:** Association of the HLA-C1 (asparagine) and C2 (lysine) group with Taiwanese AS susceptibility and clinical phenotypes.

Characteristics	HLA-C				
	C1 *	C2 *	C1C1	C1C2	C2C2
Disease					
AS	1194 (91.42%)	112 (8.58%)	542 (83%)	110 (16.85%)	1 (0.15%)
Normal	1634 (85.82%)	270 (14.18%)	703 (73.84%)	228 (23.95%)	21 (2.21%)
*p*-value	1.8441 × 10^−6^		0.0066	0.02457	-
OR (95% CI)	1.761 (1.396–2.222)		16.159 (2.171–120.294)	10.112 (1.345–76.014)	-
Sex					
Male	1958 (88.76%)	248 (11.24%)	866 (78.51%)	226 (20.49%)	11 (1%)
Female	870 (86.65%)	134 (13.35%)	379 (75.5%)	112 (22.31%)	11 (2.19%)
*p*-value	0.0881		0.0551	0.1121	-
OR (95% CI)	1.216 (0.971–1.523)		2.285 (0.982–5.316)	2.018 (0.849–4.796)	-
Syndesmophyte					
Positive	572 (92.86%)	44 (7.14%)	265 (86.04%)	42 (13.64%)	1 (0.32%)
Negative	622 (90.14%)	68 (9.86%)	277 (80.29%)	68 (19.71%)	0 (0%)
*p*-value	0.0818		0.9669	0.9657	-
OR (95% CI)	1.421 (0.957–2.111)		Not Available (N/A)	N/A	-
HLA-B27					
Positive	1103 (91.76%)	99 (8.24%)	503 (83.69%)	97 (16.14%)	1 (0.17%)
Negative	91 (87.5%)	13 (12.5%)	39 (75%)	13 (25%)	0 (0%)
*p*-value	0.1395		0.9677	0.9655	-
OR (95% CI)	1.592 (0.859–2.948)		N/A	N/A	-
Age onset					
≤16	181 (93.3%)	13 (6.7%)	84 (86.6%)	13 (13.4%)	0 (0%)
>16	2647 (87.77%)	369 (12.23%)	1161 (76.99%)	325 (21.55%)	22 (1.46%)
*p*-value	0.0234		0.9586	0.9608	-
OR (95% CI)	0.515 (0.29–0.914)		N/A	N/A	-

* HLA-C epitope.

**Table 4:** Association of the *KIRs* with Taiwanese AS susceptibility and clinical phenotypes.

Characteristics	*Killer Immunoglobulin-like Receptors (KIRs)*								
	*2DS1	*2DS2*	*2DS3*	*2DS4 del*	*2DS4 ful*	*2DS5*	*3DS1*	2DL2	2DL5
Disease									
AS	218 (33.38%)	160 (24.5%)	134 (20.52%)	353 (54.06%)	507 (77.64%)	143 (21.9%)	225 (34.46%)	176 (26.95%)	256 (39.2%)
Normal	352 (36.97%)	219 (23%)	202 (21.22%)	466 (48.95%)	743 (78.05%)	235 (24.68%)	361 (37.92%)	231 (24.26%)	441 (46.32%)
*p*-value	0.1399	0.4876	0.7363	0.0444	0.8477	0.1965	0.1569	0.2242	0.0047
*p*-value (FDR)	0.3363	0.626914286	0.8283375	0.1998	0.8477	0.3363	0.3363	0.3363	0.0423
OR (95% CI)	0.85 (0.69–1.05)	1.09 (0.86–1.37)	0.96 (0.75–1.23)	1.23 (1.01–1.5)	0.98 (0.77–1.24)	0.86 (0.68–1.08)	0.86 (0.7–1.06)	1.15 (0.92–1.45)	0.75 (0.61–0.91)
Sex									
Male	379 (34.36%)	261 (23.66%)	226 (20.49%)	569 (51.59%)	855 (77.52%)	255 (23.12%)	401 (36.36%)	277 (25.11%)	480 (43.52%)
Female	191 (38.05%)	118 (23.51%)	110 (21.91%)	250 (49.8%)	395 (78.69%)	123 (24.5%)	185 (36.85%)	130 (25.9%)	217 (43.23%)
*p*-value	0.1526	0.9454	0.5161	0.5070	0.6008	0.5449	0.8477	0.7372	0.9133
*p*-value Characteristic	0.9454	0.9454	0.9454	0.9454	0.9454	0.9454	0.9454	0.9454	0.9454
OR (95% CI)	0.85 (0.68–1.06)	1.01 (0.79–1.29)	0.92 (0.71–1.19)	1.07 (0.87–1.33)	0.93 (0.72–1.21)	0.93 (0.72–1.19)	0.98 (0.79–1.22)	0.96 (0.75–1.22)	1.01 (0.82–1.25)
Syndesmophyte									
Positive	101 (32.79%)	68 (22.08%)	56 (18.18%)	160 (51.95%)	234 (75.97%)	69 (22.4%)	106 (34.42%)	74 (24.03%)	116 (37.66%)
Negative	117 (33.91%)	92 (26.67%)	78 (22.61%)	193 (55.94%)	273 (79.13%)	74 (21.45%)	119 (34.49%)	102 (29.57%)	140 (40.58%)
*p*-value	0.7619	0.1740	0.1627	0.3067	0.3334	0.7686	0.9835	0.1118	0.4465
*p*-value (FDR)	0.864675	0.522	0.522	0.60012	0.60012	0.864675	0.9835	0.522	0.66975
OR (95% CI)	0.95 (0.69–1.32)	0.78 (0.54–1.12)	0.76 (0.52–1.12)	0.85 (0.63–1.16)	0.83 (0.58–1.21)	1.06 (0.73–1.53)	1 (0.72–1.38)	0.75 (0.53–1.07)	0.88 (0.65–1.21)
B27									
Positive	196 (32.61%)	144 (23.96%)	118 (19.63%)	326 (54.24%)	468 (77.87%)	132 (21.96%)	202 (33.61%)	158 (26.29%)	231 (38.44%)
Negative	22 (42.31%)	16 (30.77%)	16 (30.77%)	27 (51.92%)	39 (75%)	11 (21.15%)	23 (44.23%)	18 (34.62%)	25 (48.08%)
*p*-value	0.1573	0.2749	0.0595	0.7475	0.6339	0.8923	0.1246	0.1966	0.1739
*p*-value (FDR)	0.35388	0.41235	0.35388	0.8409375	0.815014286	0.8923	0.35388	0.35388	0.35388
OR (95% CI)	0.66 (0.37–1.17)	0.71 (0.38–1.31)	0.55 (0.3–1.02)	1.1 (0.62–1.94)	1.17 (0.61–2.26)	1.05 (0.52–2.1)	0.64 (0.36–1.13)	0.67 (0.37–1.23)	0.67 (0.38–1.19)
Age onset									
≤16	27 (28.72%)	28 (29.79%)	21 (22.34%)	51 (54.26%))	77 (81.91%)	16 (17.02%)	26 (27.66%)	29 (30.85%)	36 (38.3%))
>16	191 (34.17%)	132 (23.61%)	113 (20.21%)	302 (54.03%)	430 (76.92%)	127 (22.72%)	199 (35.6%)	147 (26.3%)	220 (39.36%)
*p*-value	0.1598	0.1344	0.6630	0.4633	0.3844	0.1514	0.0689	0.1244	0.3841
*p*-value (FDR)	0.28764	0.28764	0.663	0.5212125	0.494228571	0.28764	0.28764	0.28764	0.494228571
OR (95% CI)	1.38 (0.88–2.17)	0.71 (0.45–1.11)	0.9 (0.55–1.47)	0.86 (0.57–1.29)	0.79 (0.47–1.34)	1.48 (0.87–2.53)	1.53 (0.97–2.41)	0.71 (0.45–1.1)	1.21 (0.79–1.83)

*2DS1: Two Ig Domains and Short cytoplasmic tail 1.

**Table 5:** Association of the KIR and HLA-C combinations with Taiwanese AS susceptibility.

KIR	HLA-C	AS	Normal	*p*-Value	*p*-Value (FDR)	OR (95% CI)
2DS1	C1C1	184 (28.18%)	265 (27.84%)	0.8809	0.99	1.02 (0.81–1.27)
2DS1	C1C2	33 (5.05%)	82 (8.61%)	0.0073	0.160867	0.56 (0.37–0.86)
2DS1	C2C2	1 (0.15%)	5 (0.53%)	0.2597	0.597127	0.29 (0.03–2.49)
2DS2	C1C1	132 (20.21%)	159 (16.7%)	0.0731	0.386821	1.26 (0.98–1.63)
2DS2	C1C2	28 (4.29%)	56 (5.88%)	0.1605	0.509588	0.72 (0.45–1.14)
2DS2	C2C2	0 (0%)	4 (0.42%)	0.9728	0.99	0.00 (0.00–I)
2DS3	C1C1	113 (17.3%)	147 (15.44%)	0.3198	0.636137	1.15 (0.88–1.50)
2DS3	C1C2	21 (3.22%)	51 (5.36%)	0.0440	0.295965	0.59 (0.35–0.99)
2DS3	C2C2	0 (0%)	4 (0.42%)	0.9728	0.99	0.00 (0.00–I)
2DS4del	C1C1	296 (45.33%)	342 (35.92%)	0.0002	0.0127	1.48 (1.21–1.81)
2DS4del	C1C2	57 (8.73%)	112 (11.76%)	0.0524	0.295965	0.72 (0.51–1.00)
2DS4del	C2C2	0 (0%)	12 (1.26%)	0.9692	0.99	0.00 (0.00–8.45 × 10^299^)
2DS4ful	C1C1	416 (63.71%)	550 (57.77%)	0.0172	0.198582	1.28 (1.05–1.57)
2DS4ful	C1C2	90 (13.78%)	176 (18.49%)	0.0131	0.1905	0.70 (0.53–0.93)
2DS4ful	C2C2	1 (0.15%)	17 (1.79%)	0.0164	0.198582	0.08 (0.01–0.64)
2DS5	C1C1	120 (18.38%)	181 (19.01%)	0.7491	0.99	0.96 (0.74–1.24)
2DS5	C1C2	22 (3.37%)	52 (5.46%)	0.0517	0.295965	0.60 (0.36–1.00)
2DS5	C2C2	1 (0.15%)	2 (0.21%)	0.7961	0.99	0.73 (0.07–8.05)
3DS1	C1C1	187 (28.64%)	270 (28.36%)	0.9042	0.99	1.01 (0.81–1.26)
3DS1	C1C2	37 (5.67%)	86 (9.03%)	0.0135	0.1905	0.60 (0.41–0.90)
3DS1	C2C2	1 (0.15%)	5 (0.53%)	0.2597	0.597127	0.29 (0.03–2.49)
2DL2	C1C1	146 (22.36%)	165 (17.33%)	0.0125	0.1905	1.37 (1.07–1.76)
2DL2	C1C2	30 (4.59%)	61 (6.41%)	0.1245	0.489324	0.70 (0.45–1.10)
2DL2	C2C2	0 (0%)	5 (0.53%)	0.9696	0.99	0.00 (0.00–7.85 × 10^281^)
2DL5	C1C1	216 (33.08%)	323 (33.93%)	0.7234	0.967072	0.96 (0.78–1.19)
2DL5	C1C2	39 (5.97%)	111 (11.66%)	0.0002	0.0127	0.48 (0.33–0.70)
2DL5	C2C2	1 (0.15%)	7 (0.74%)	0.1413	0.505531	0.21 (0.03–1.69)

## Supplementary Materials

Supplementary materials can be found at www.mdpi.com/1422-0067/18/8/1775/s1.

## Abbreviations

AS	Ankylosing Spondylitis
SpA	Spondyloarthritis
NK	Natural Killer
HLA	Human Leukocyte Antigen
KIR	Killer immunoglobulin-like receptor
FLS	Fibroblast-like synoviocytes
MMPs	Matrix metalloproteinases
RA	Rheumatoid arthritis
mSASSS	Modified Stoke’s Ankylosing Spondylitis Spinal Score
pVIPR	Vasoactive intestinal peptide receptor type 1
DOPE	Discrete Optimized Protein Energy
FDR	False discovery rate
HCMV	Human cytomegalovirus
SLE	Systemic lupus erythaematosus
