# Supplementary material for: Human Leukocyte Antigen C*12:02:02 and Killer Immunoglobulin-Like Receptor 2DL5 are Distinctly Associated with Ankylosing Spondylitis in the Taiwanese
Source: Int J Mol Sci. 2017 Aug 16;18(8):1775. doi: 10.3390/ijms18081775 (PMC5578164; doi:10.3390/ijms18081775)
Supplement: Supplementary file 1 [file ijms-18-01775-s001.pdf]

Figure S1

```
                                               1  α1-α2 domains
C*01:02:01    MRVMAPRTLI LLLSGALALT ETWA------ CSHSMKYFFT SVSRPGRGEP RFISVGYVDD TQFVRFDSDA ASPRGEPRAP WVEQEGPEYW DRETQKYKRQ  70
C*03:02:01    ---------- ---------- ---------- G----R--Y- A--------- H--A------ ---------- ---------- ---------- ----------
C*12:02:02    ---------- ---------- ---------- -----R--Y- A--------- ---A------ ---------- ---------- ---------- ----------

C*01:02:01    AQTDRVSLRN LRGYYNQSEA GSHTLQWMCG CDLGPDGRLL RGYDQYAYDG KDYIALNEDL RSWTAADTAA QITQRKWEAA REAEQRRAYL EGTCVEWLRR  170
C*03:02:01    ---------- ---------- ---I--R-Y- --V------- -----S---- ---------- ---------- ---------- -----L---- --L-------
C*12:02:02    --A------- ---------- ------R-Y- ---------- -----S---- ---------- ---------- ---------- -----W---- ----------

                              α3 domain
C*01:02:01    YLENGKETLQ RAEHPKTHVT HHPVSDHEAT LRCWALGFYP AEITLTWQWD GEDQTQDTEL VETRPAGDGT FQKWAAVMVP SGEEQRYTCH VQHEGLPEPL  270
C*03:02:01    --K------- ---------- ---------- ---------- ---------- ---------- ---------- -------V-- ---------- ----------
C*12:02:02    ---------- ---------- ---------- ---------- --------R- ---------- ---------- -------V-- ---------- ----------

                         Transmembrane helix
C*01:02:01    TLRWEPSSQP TIPIVGIVAG LAVLAVLAVL GAVVAVVMCR RKSSGGKGGS CSQAASSNSA QGSDESLIAC KA  342
C*03:02:01    ---------- ---------- ---------- ---------- ---------- ---------- ---------- --
C*12:02:02    ---------- ---------- ---------- ---M------ ---------- ---------- ---------- --
```

Fig. S1. The sequence alignment of HLA-C*12:02:02 with HLA-C*01:02:01 and HLA-C*03:02:01. The amino-acid sequences, residue numbers and the sequence alignment of HLA-C alleles were searched from the website of HLA Nomenclature (http://hla.alleles.org). The three HLA-C alleles are proteins of 342 amino acids, consisting of α1–α2 domains (yellow region), α3 domain (green region) and a transmembrane helix (cyan region). The amino-acid residues marked in magenta represent the unique amino-acid residues in the HLA-C*12:02:02, when compared to the sequence of HLA-C*01:02:01 and HLA-C*03:02:01.
